# Transcriptomics analysis revealing candidate genes and networks for sex differentiation of yesso scallop (*Patinopecten yessoensis*)

**DOI:** 10.1186/s12864-019-6021-6

**Published:** 2019-08-23

**Authors:** Liqing Zhou, Zhihong Liu, Yinghui Dong, Xiujun Sun, Biao Wu, Tao Yu, Yanxin Zheng, Aiguo Yang, Qing Zhao, Dan Zhao

**Affiliations:** 1 Key Laboratory for Sustainable Development of Marine Fisheries, Ministry of Agriculture, Yellow Sea Fisheries Research Institute, Chinese Academy of Fishery Science, Qingdao, China; 2 Labortory for Fisheries Science and Food Production Processes, Qingdao National Laboratory for Marine Science and Technology, Qingdao, China; 3 Zhejiang Wanli University, Ningbo, China; 4 Changdao Enhancement and Experiment Station, Chinese Academy of Fishery Science, Changdao, China; 5 College of Fisheries and Life Science, Shanghai Ocean University, Shanghai, China

**Keywords:** Yesso scallop (*Patinopecten yessoensis*), Gonad, Sex determination and differentiation, Transcriptome, Weighted gene co-expression network analysis (WGCNA)

## Abstract

**Background:**

The Yesso scallop, *Patinopecten* (*Mizuhopecten*) *yessoensis,* is a commercially important bivalve in the coastal countries of Northeast Asia. It has complex modes of sex differentiation, but knowledge of the mechanisms underlying this sex determination and differentiation is limited.

**Results:**

In this study, the gonad tissues from females and males at three developmental stages were used to investigate candidate genes and networks for sex differentiation via RNA-Req. A total of 901,980,606 high quality clean reads were obtained from 18 libraries, of which 417 expressed male-specific genes and 754 expressed female-specific genes. Totally, 10,074 genes differentially expressed in females and males were identified. Weighted gene co-expression network analysis (WGCNA) revealed that turquoise and green gene modules were significantly positively correlated with male gonads, while coral1 and black modules were significantly associated with female gonads. The most important gene for sex determination and differentiation was *Pydmrt 1*, which was the only gene discovered that determined the male sex phenotype during early gonadal differentiation. Enrichment analyses of GO terms and KEGG pathways revealed that genes involved in metabolism, genetic and environmental information processes or pathways are sex-biased. Forty-nine genes in the five modules involved in sex differentiation or determination were identified and selected to construct a gene co-expression network and a hypothesized sex differentiation pathway.

**Conclusions:**

The current study focused on screening genes of sex differentiation in Yesso scallop, highlighting the potential regulatory mechanisms of gonadal development in *P. yessoensis*. Our data suggested that WCGNA can facilitate identification of key genes for sex differentiation and determination. Using this method, a hypothesized *P. yessoensis* sex determination and differentiation pathway was constructed. In this pathway, *Pydmrt 1* may have a leading function.

**Electronic supplementary material:**

The online version of this article (10.1186/s12864-019-6021-6) contains supplementary material, which is available to authorized users.

## Background

*Patinopecten* (*Mizuhopecten*) *yessoensis* is a cold-water species distributed along the coasts of China, Korea, Japan, and Russia. Particularly in the northern provinces of China, Liaoning, and the northern edge of the Shandong peninsula, it is large in size, fetches a high market price, and has become an important scallop species since it was introduced to China from Japan in 1980 [1]. In recent years, it has been hypothesized that hermaphrodites of the species could be used to improve a variety of traits through the construction of inbred lines, however, knowledge of the mechanisms underlying sex determination and differentiation in this species is very limited, which has hindered these improvements. It is known that the existence of hermaphrodites is commonly associated with the deterioration of aquaculture environments [2], nevertheless, we do not yet understand the molecular mechanisms underlying this association. Activation of the testis or ovarian pathway, or the repression of the alternative pathway, with many genes being expressed in a sexually dimorphic manner, determines the resulting sex, while all the pathway and mechanism of sex differentiation in mollusck were not clear. Amplified fragment length polymorphism (AFLP) markers have been used by several Chinese research groups to construct genetic linkage maps for *P. yessoensis* since 2009 [3, 4]. Based on these genetic linkage maps, in a previous study we screened some sex-related AFLP molecular markers under the assumptions that hermaphroditism was an independent group and that there were differences in genetic structure between male and female individuals [5]. In recent years, some genes related to sex determination or differentiation have been identified and characterized through transcriptome analysis of male and female mature gonads. These included *Dmrt1*, *Sox9*, *fem1,* and *Vasa* [6].

Data from the transcriptome sequencing and de novo analysis of Yesso Scallop helped to pinpoint candidate genes potentially involved in growth, reproduction, stress/immunity-responses, and so on [7]. Therefore, transcriptomics tools are widely used for screening sex determination and differentiation genes in many kinds of animals including bivalves. Teaniniuraitemoana et al. carried out transcriptome analysis on several *Pinctada margaritifera* gonadic samples from males and females at different development stages to identify potential sex differentiation and sex determining genes, such as *dmrt* and *fem-1* for males, and *foxl2* and vitellogenin for females [8]. The transcriptomes of three ovaries and three testes of Yesso scallop were sequenced and analyzed by Li et al., and the results showed that *PyFOXL2* was ovary-biased, and that *PyDMRT* and *PySOXH* were testis-biased, the three genes were presumed to be key candidates for scallop sex determination/differentiation [9]. Sex-related genes, such as *forkhead box L2* (*foxl2*), sex determining region Y-box (*Sox*), beta-catenin (*β-catenin*), chromobox homolog (*CBX*), and Sex-lethal (*Sxl*) were also identified from the transcriptome data of gonads obtained from blood clams (*Tegillarca granosa*) [10]. As a correlation-based method that describes and visualizes networks of data points using the R software package [11], and an analysis method based on a large sample size of transcription group data, weighted gene correlation network analysis (WGCNA) can quickly identify core sex-related pathways and genes that play an important role in transcription regulation, and easily predict unknown gene regulatory relationships through known genes in the network. Yue et al. found that *CgDsx* and *CgFoxl* participate in the pathway of Pacific oyster sex determination, with *CgDsx* expressed more in males and *CgFoxl* expressed more in females, particularly during early gonad development [12]. To gain insight into the molecular mechanisms underlying female-superior sexual dimorphism with respect to growth rate, WGCNA analysis of female banana shrimp (*Fenneropenaeus merguiensis*) digestive gland tissue samples was completed by Powell et al. and revealed a gene module containing a large number of transcripts with homology to transcription factors and genes associated with growth regulation [13]. Wang et al. found two gene modules (yellowgreen and salmon4) that were significantly positively correlated with female-biased sexual size dimorphism using WGCNA, and identified a series of hub genes by drawing an illustrated network map of these two modules in Chinese tongue sole (*Cynoglossus semilaevis*) [14].

Although the genomes between the sexes are largely similar, differences in gene expression in gonads can also be due to long noncoding RNA (lincRNA) [15] and other agents such as genetic sex determination (GSD) and environmental sex determination (ESD) factors. It is difficult to identify genes correlated with sex determination or differentiation through conventional RNA-Seq analysis. In the present study, we aimed to identify genes that may be important for sex differentiation by investigating transcriptional changes in Yesso scallop female and male individuals at three gonad development stages (mature, immature, and spawning) using WGCNA, based on the *Mizuhopecten yessoensis* genome data released on the NCBI (National Center for Biotechnology Information).

## Results

### Overall transcriptome and sequencing data

To better understand *P. yessoensis* sex determination and differentiation mechanisms, we conducted a comparative transcriptomic analysis. A total of 18 libraries were constructed and named as follows: PyUmf-1 to 3, PyUmm-1 to 3, PyMf-1 to 3, PyMm-1 to 3, PyOf-1 to 3, and PySm-1 to 3. Gonad development stage was identified via histological methods, and hermaphrodites were excluded. Unfortunately, one of the four individual gonad development stages of PySm-1 was not accurately defined, which affect the trends of overall data, therefore, we excluded the data of PySm-1. As a result, a mean of 50,354,447 filtered clean reads with Q20 above 97.16% was obtained from each library and 1.55–2.75% of low quality reads mapped with rRNA were removed.

### Identification of DEGs

**Fig. 1:**
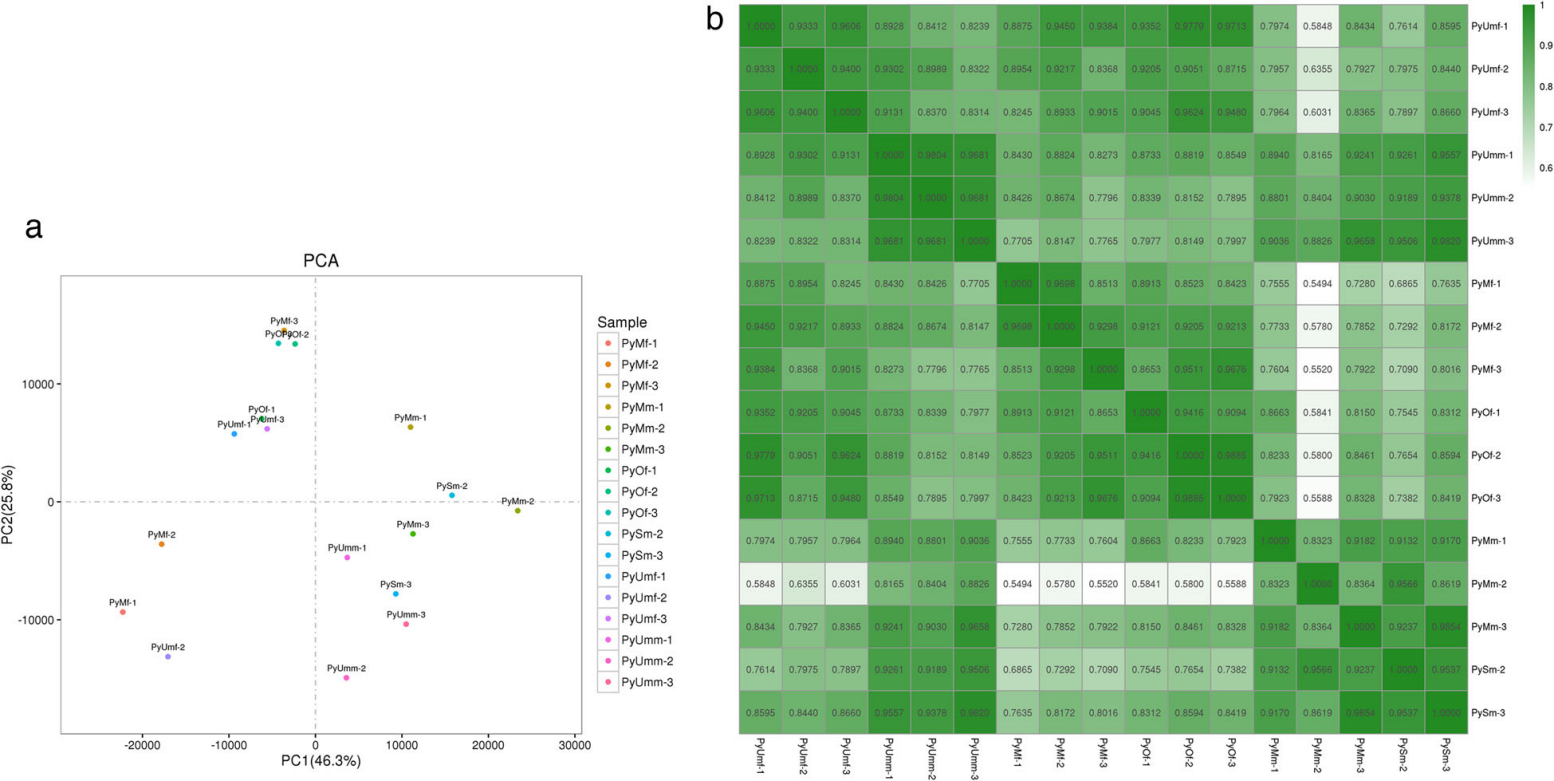
**a** Principal components analysis reveal strong clustering associated with sex (PC1 accounted for 44% of the variance). **b** Sample relationship heatmap plot revealed exact sex identification except for PySm-1. Dark green represents strong correlation and light green represents weak correlation, each column and row corresponds to one sample’s relationships with the other 18 samples including itself

Principal components analysis revealed strong clustering associated with sex, excluding sample PySm-1 (Fig. 1a). PC1 accounted for 44% of the differential expression and was correlated with sex. However, different development stages did not cluster obviously, as PC2 accounted for only 24.4% of the differential expression. In the sample relationship analysis, a heatmap plot revealed that sex identification of samples was exact except for the PySm-1 group (Fig. 1b). Therefore, we did not include the RNA-Seq data of sample PySm-1 in later analyses.

**Fig. 2:**
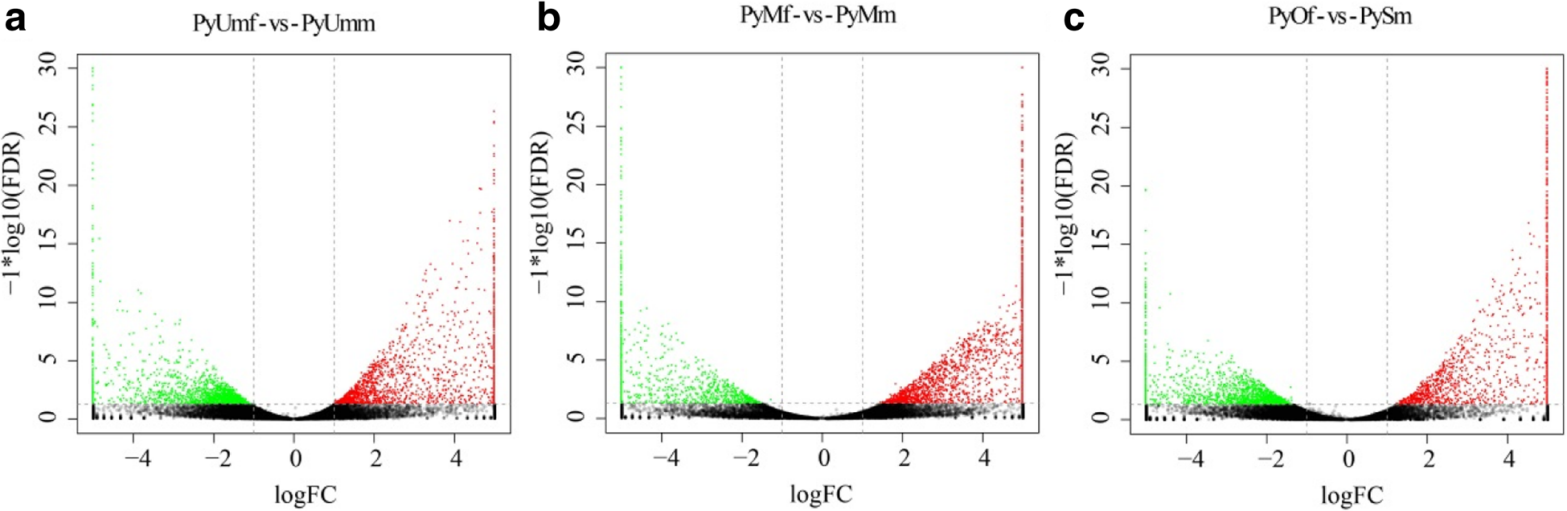
DEGs identified from six gonad groups. Red indicates upregulated genes, and green indicates downregulated genes among the six gonad groups

In the differential expression analysis, 3412 genes in PyUmf-vs-PyUmm, 2909 genes in PyOf-vs-PySm, and 2778 genes in PyMf-vs-PyMm were found to differentiate between females and males. Fewer differential genes were found between PyUmf and PyMf, PyMf and PyOf, PyUmf and PyOf, PyUmm and PySm, PyUmm and PyMm, and between PyMm and PySm, with 1504, 920, 381, 249, 42, and 14 differential genes, respectively (fold change ≥2, *P* < 0.05). DEGs were very abundant between females and males (Fig. 2). However, there were fewer DEGs between different gonad development stages.

Subsequently, a total of 23,292 known genes and 2237 novel genes were identified via transcript reconstruction of the *P. yessoensis* genome. Of the 25,529 protein coding genes analyzed, 1171 showed significant differences between female and male gonads, and there were more female-specific genes (754) than male-specific genes (417) (Additional file 1**:** Figure S1).

### Gene co-expression network interactions differ between the sexes

**Fig. 3:**
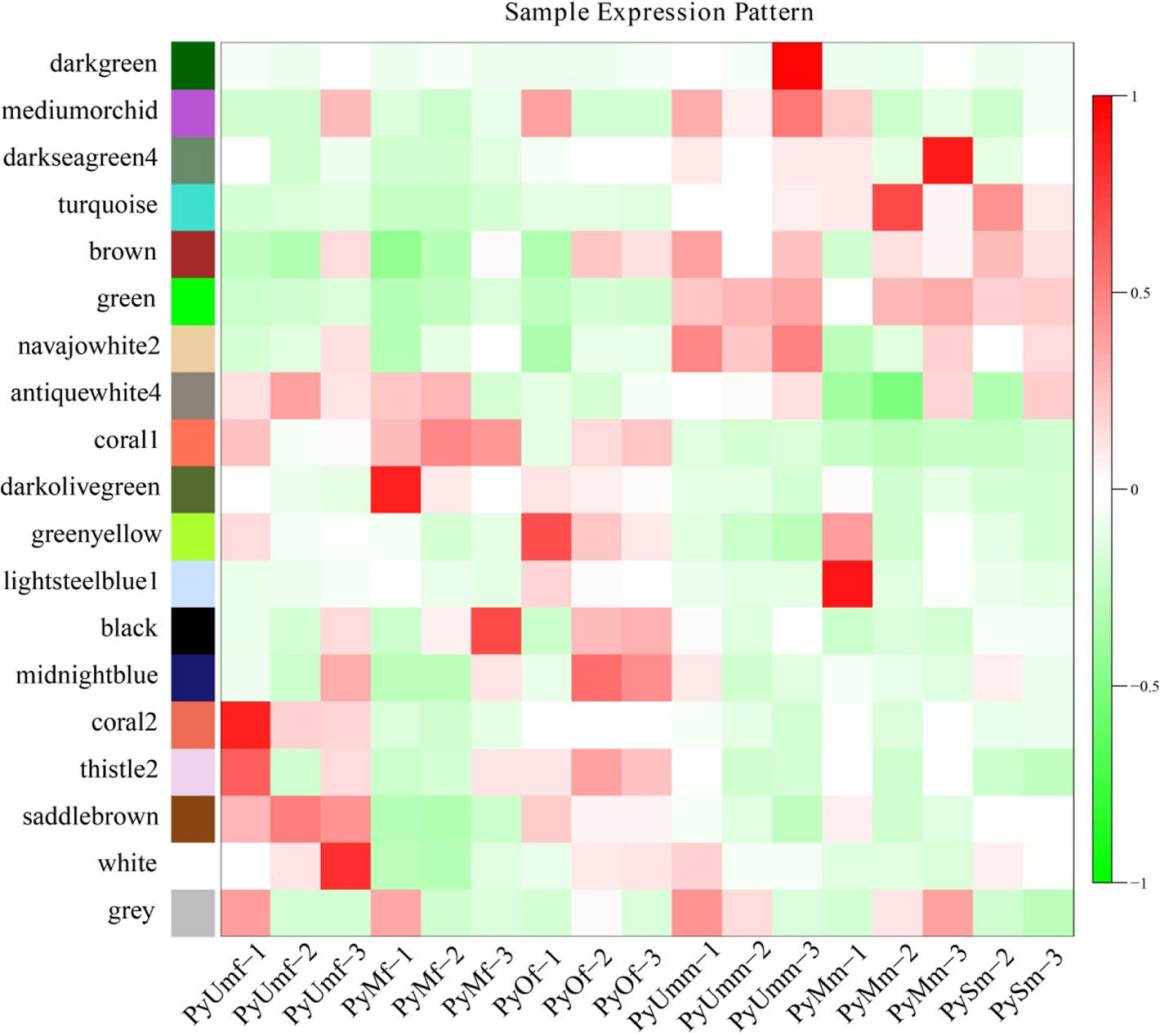
Heatmap of samples expression patterns. The expression patterns of 19 modules including darkgreen, mediumorchid, darkseagreen4, turquoise, brown, green, navajowhite2, antiquewhite4, coral1, darkolivegreen, greenyellow, lightsteelblue1, black, midnightblue, coral2, thistle2, saddlebrown, white, and grey are shown as a heatmap. The color bar indicates expression levels from high (red) to low (green)

WGCNA revealed that the genes of female and male scallop gonads can be clustered into 19 modules (Fig. 3), which were further classified and clustered by similarity = 0.75 and minModuleSize = 50, with module sizes ranging from 79 to 4058. Of these modules, the turquoise module with size 1541 and the green module with size 1451 were significantly positively correlated with male gonads and were negatively correlated with female gonads. In contrast, the coral1 module with size 1371 and the black module with size 860 were significantly positively correlated with female gonads and were negatively correlated with male gonads.

### Functional enrichment analysis of the genes

**Fig. 4:**
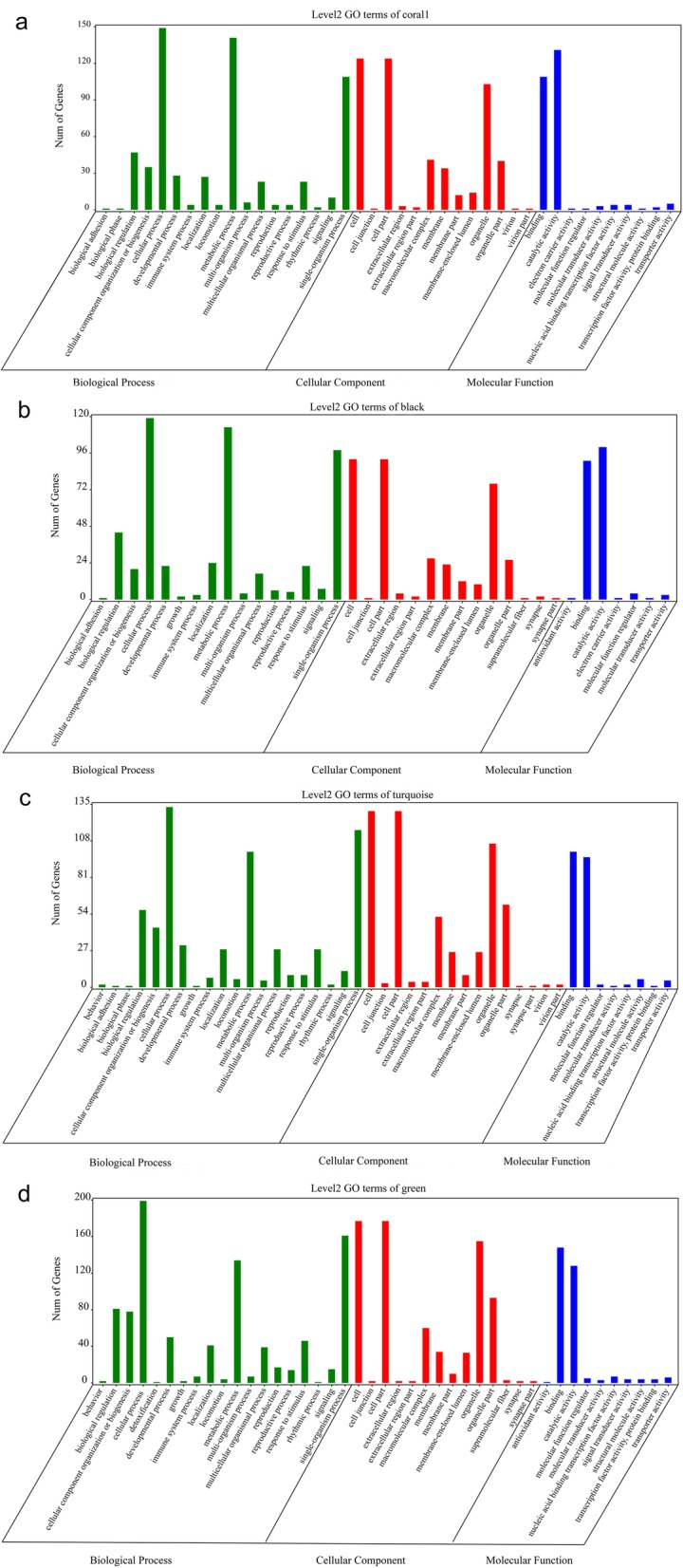
Gene ontology term enrichment in four modules of RNA-Seq

**Fig. 5:**
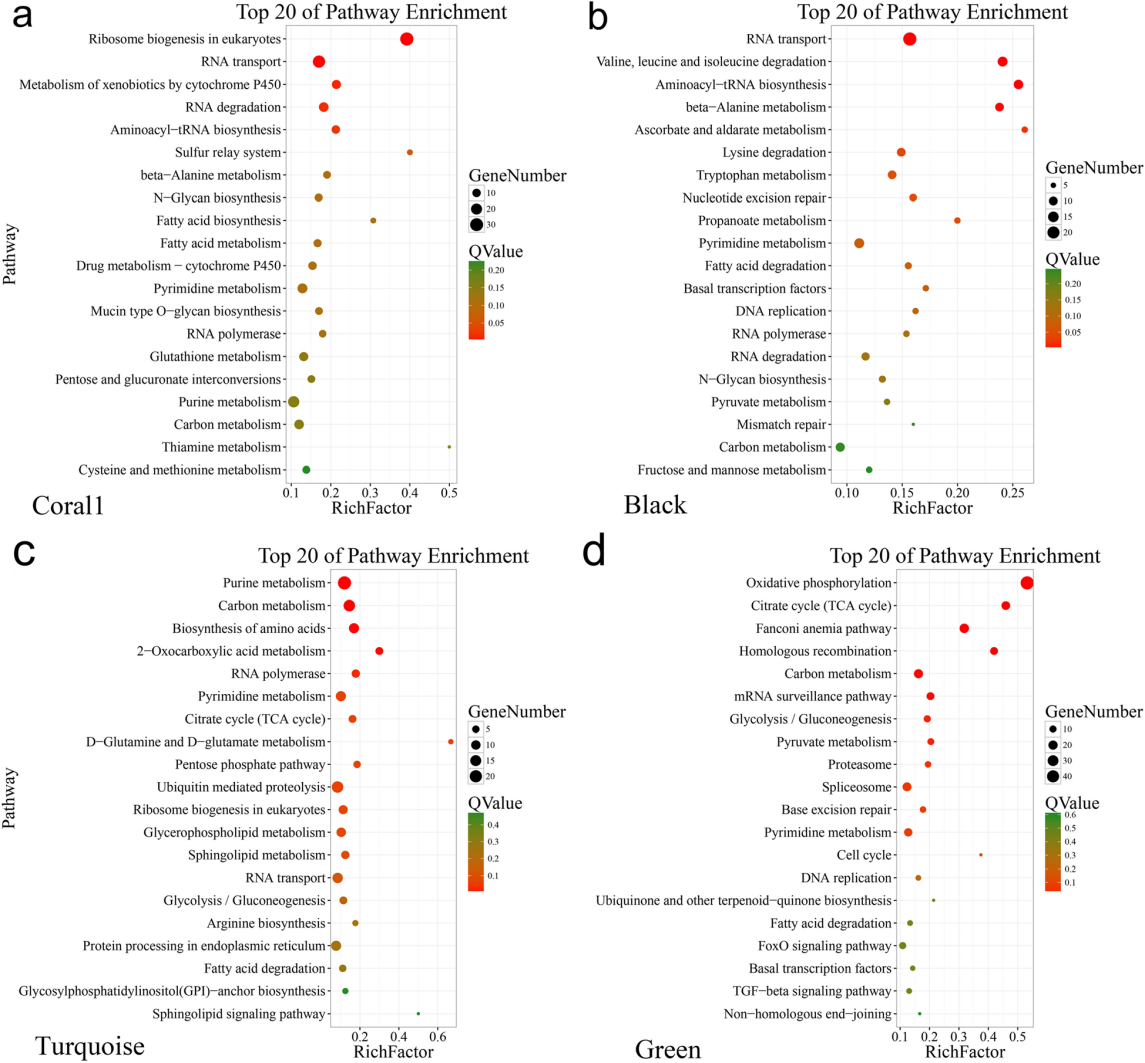
KEGG functional enrichment of differentially expressed genes (DEGs) in the coral1, black, turquoise and green modules generated by hierarchical clustering of WGCNA. Rich Factor is the ratio of the number of genes in the specific module to the number of genes annotated in the pathway. The greater the Rich Factor, the greater the degree of enrichment

To better understand the function of these differentially expressed genes in sex differentiation or determination, GO term enrichment (Fig. 4) and KEGG pathway enrichment were conducted. Seventy-eight GO terms (belonging to biological processes, cellular components, and molecular functions) with *p* < 0.05 were significantly enriched in the coral1 module, 78 GO terms were enriched in the black module, 160 terms were significantly enriched in the turquoise module, and 237 were enriched in the green module. Most were enriched as cellular processes, metabolic processes, or single-organism progresses, which can all be classified as biological processes. Lots were enriched as cells, cell parts, or organelles, which areclassified under cellular components. Lots of terms were also enriched as binding or catalytic activities, which can be categorized as molecular functions. At least 49 genes in five modules were identified as being involved in sex differentiation or determination (Additional file 2: Table S1), and most of these were enriched in the GO function terms mentioned above. In total, out of all of the transcripts, 5030 genes had KEGG assignations. These mainly belonged to metabolic processes, environmental information processing functions, and genetic information processing pathways. More specifically, ribosome biogenesis in eukaryotes, RNA transport, metabolism of xenobiotics by cytochrome P450, degradation of valine, leucine and isoleucine, aminoacyl-tRNA biosynthesis, and beta-Alanine metabolism were significantly enriched in females (Fig. 5a, b), and purine metabolism, carbon metabolism, biosynthesis of amino acids, oxidative phosphorylation, the TCA cycle, and the fanconi anemia pathway were significantly enriched in males (Fig. 5c, d). All of these GO term enrichments and KEGG pathway enrichments were closely related to sex differentiation or determination. Some of them belonged to cellular process pathways, which are relevant to gamete development.

### Quantitative real-time PCR

**Fig. 6:**
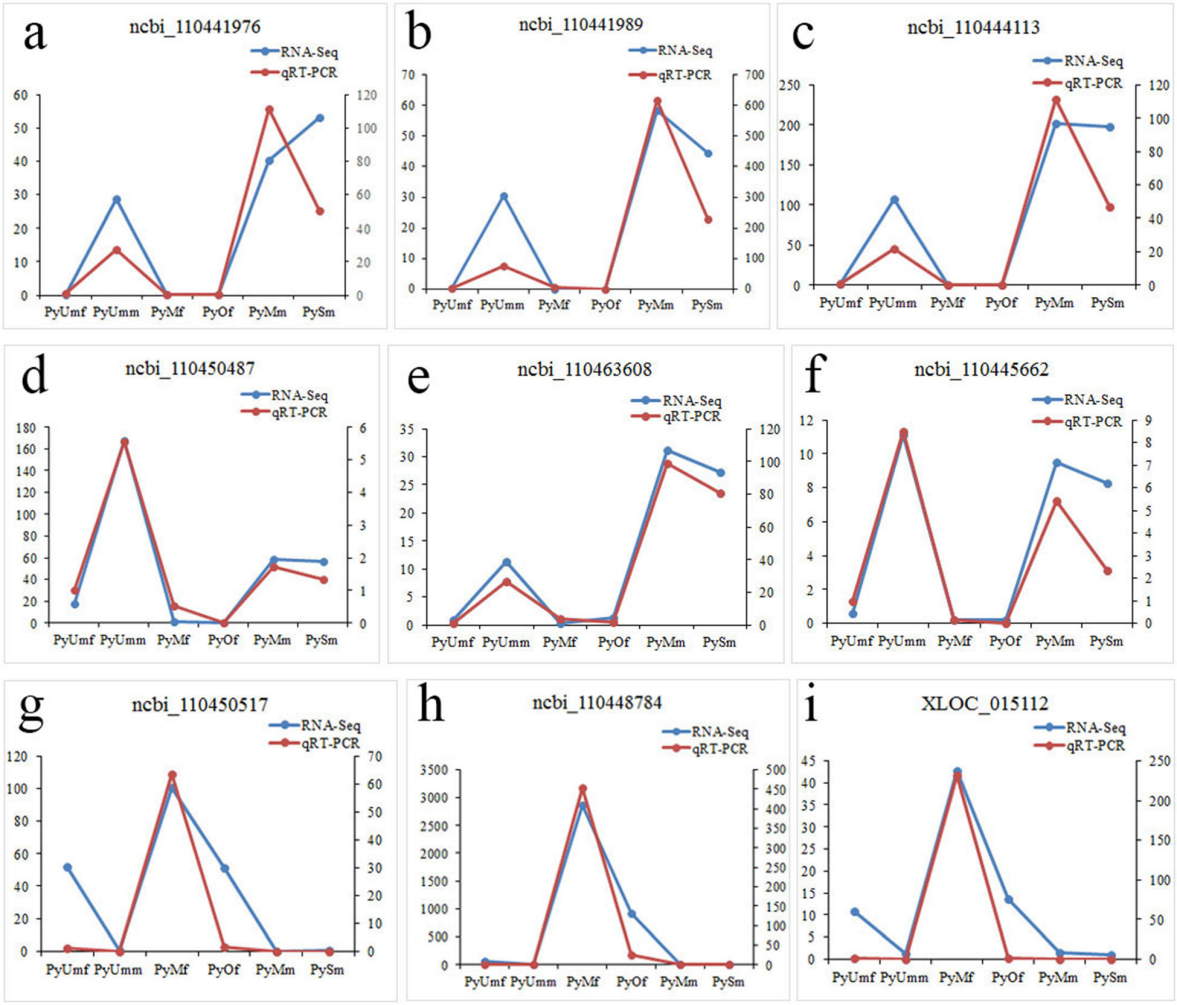
Quantitative real-time PCR analysis of nine sex-biased genes in male and female gonads of *P. yessoensis*. The relative mRNA expressions of the nine genes were calculated using the 2^-△△Ct^ method, with β-actin as a reference gene. The vertical axis scale on the left corresponds to the RNA-Seq value, and the vertical axis scale on the right corresponds to the qRT-PCR value

Fifteen sex-biased genes were randomly selected for qRT-PCR to validate differential expressions identified by RNA-Seq. Comparing the transcriptome data with the qRT-PCR results, we found that the patterns of transcript abundance detected for genes using qRT-PCR were consistent with the RNA-Seq results (Fig. 6). The expression pattern of ncbi_110450487(*Dmrt 1*) (Fig. 6d) was different from the expression patterns of the male-biased genes shown in Figs. 6a, b, c, e, and f. Its expression was also remarkably different from the expressions of the female-biased genes shown in Figs. 6g, h, and i. Therefore, *Pydmrt 1* was found to be the only one gene which determined the male sex phenotype during early gonadal differentiation in darkgreen module.

### Identification of sex-biased hub genes and network construction

In the turquoise module, the genes in the core positions were ncbi_110441989 (*cAMP and cAMP-inhibited cGMP 3′,5′-cyclic phosphodiesterase 10A*, *PDE*), ncbi_110442454 (*testis-specific serine/threonine-protein kinase 3 –like*, *tssk-3*), ncbi_110466991 (*WD repeat-containing protein on Y chromosome-like*, *WD rcp*), and ncbi_110444113 (*leucine-rich repeat-containing protein*, *LRR*) (Additional file 3: Figure S2). In the green module, ncbi_110443603 (*choline transporter-like protein 1*, *CTL1*), ncbi_110465229 (*actin 5C*), and ncbi_110441893 (*basic helix-loop-helix and HMG box domain- containing protein 1*, *bHLH*) were in the core positions (Additional file 4: Figure S3). Other genes associated with these genes all had the same expression patterns, and were much more highly expressed in male gonads than in female gonads.

In the coral1 module, ncbi_110465748 (*polypeptide N-acetylgalactosaminyltransferase 4*, *GALNT4*), ncbi_110454866 (*protein ovo-like isoform X4*), ncbi_110450517 (Cytochrome P450 1A4-like, *CYP1A4-like*), ncbi_110450286(*forkhead box protein A2-A-like*, *fox A2-like*), and XLOC_015112 (*transcriptional regulatory protein TBS1-like*, *TBS1-like*) were in the core positions (Additional file 5: Figure S4). In the black module, ncbi_110456621 (*glycoprotein 3-alpha-L- fucosyltransferase A*, *Fuc-T*), ncbi_110447244 (*collagen alpha-2(I) chain*), ncbi_110440232 (uncharacterized protein LOC105336037), and ncbi_110448784(*Vitellogenin*, *Vg*) were in the core positions (Additional file 6: Figure S5). Other genes associated with these genes all had the same expression patterns, and were much more highly expressed in female gonads than in male gonads.

**Fig. 7:**
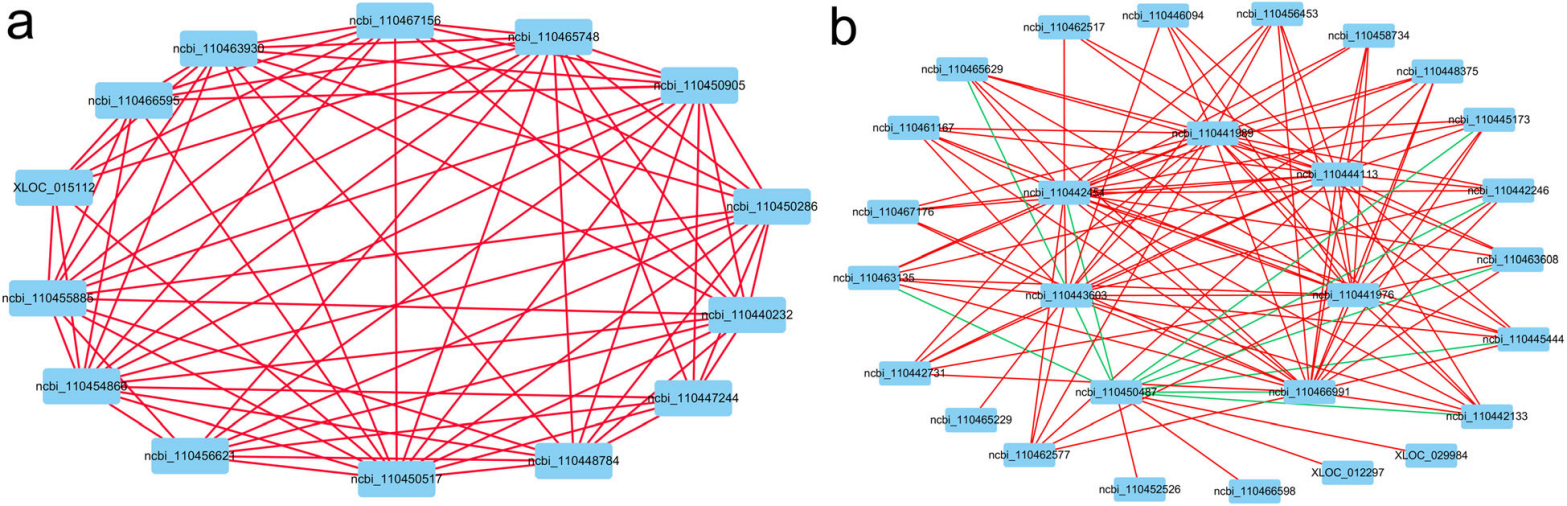
The relationships between the 49 selected genes separated into female and male groups. Red lines indicate a positive relationship between two genes, green lines indicate a negative relationship between two genes. **a** Network constructed based on female gonad RNA-Seq data. **b** Network constructed based on male gonad RNA-Seq data, the negative hub gene was ncbi_110450487 (*dmrt 1*)

The 49 selected genes were used to construct networks based on their relationship coefficients of expression mode in female and male groups (Fig. 7). In the female group, ncbi_110450517(*CYP1A4-like*), ncbi_110465748(*GALKNT4*) and ncbi_110454866(*protein ovo-like*) are the hub genes, other genes, such as ncbi_110447244 (*collagen alpha-2(I) chain*), XLOC_015112 (*TBS1-like*) and ncbi_110456621 (*Fuc-T*) are positively related to these hub genes. In the male group, ncbi_11450487 (*dmrt 1*) was a negative hub gene, while ncbi_110441989 (*PDE*), ncbi_110444113 (*LRR*), ncbi_110441976 (*sox 30*), ncbi_110466991 (*WD rcp*), ncbi_110443603 (*CTL 1*) and ncbi_110442454 (tssk) were all positive hub genes, ncbi_110463608 (*RFX4*), ncbi_110463135 (*IF-2-like*), ncbi_110442133 (*MARCH3*), ncbi_110442246 (*stabilizer of axonemal microtubules*, *MTs*), ncbi_110445444(*Ras guanine nucleotide*) and ncbi_110445173(*Biorientation of chromosomes in cell division protein 1*) were closely negatively related to ncbi_11450487 (*dmrt 1*), but positively related to other hub genes.

### Hypothesized sex differentiation pathway in *Patinopecten yessoensis*

**Fig. 8:**
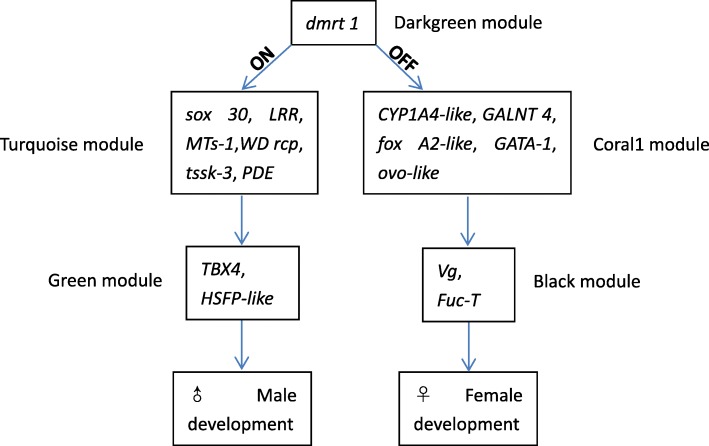
Hypothesized sex determination and differentiation pathway in *P. yessoensis*

By analyzing the overall gene expression profiles of gonads, at least 49 genes in five modules involved in sex differentiation or determination were identified (Additional file 2:Table S1). This study found that *dmrt 1* was in the darkgreen module, and that its expression was much higher in PyUmm than in other male stages, while very low or none in all female gonads. This expression trend was consistent both in q RT-PCR and de novo analysis (Fig. 6), which means that high expression of *Pydmrt 1* determines the male sex phenotype during early gonadal differentiation. Further studies should focus on validating the function of *dmrt 1*. Other selected target genes were identified as transcription factors (TFs), and they were enriched in males or females by comparing all the datasets, especially the correlation coefficients, expression levels, and gonad developmental stage. Therefore, we propose that *dmrt 1* may have a leading function in the sex differentiation pathway of *P. yessoensis*, and that significantly high expression of *dmrt 1* directly activates *sox 30* (*transcription factor SOX-30*), *LRR* (*leucine-rich repeat-containing protein*), *MTs*, *WD rcp* (*WD repeat-containing protein on Y chromosome*), *tssk-3* (*testis-specific serine/threonine-protein kinase 3*), and *cAMP and cAMP-inhibited cGMP 3′,5′-cyclic phosphodiesterase* (*PDE*), which were all male-specific genes in the turquoise module. *TBX4* (*T-box transcription factor 4*), *CTL 1*(*choline transporter-like protein 1*), *Protein B4*, and *HSFP-like* (*heat shock factor protein-like*) were in the green module and were highly expressed genes. On the other hand, *CYP1 A4-like* (*Cytochrome P450 1A4-like*), *GALNT 4* (*polypeptide N-acetylgalactosaminyltransferase 4*), *fox A2-like* (*forkhead box protein A2- like*), *GATA-1*, and *ovo-like* were in the coral1 module and were female-specific genes. *Vg* (*Vitellogenin*) and *Fuc-T* (*glycoprotein 3-alpha-L- fucosyltransferase A*) were high expression genes in the black module. Therefore, we propose the following sex differentiation pathway in *P. yessoensis* (Fig. 8):

## Discussion

By analyzing the overall gene expression profiles of scallop gonads, at least 49 genes in five modules involved in sex differentiation or determination were identified. These included *dmrt 1*, *sox 30*, *RFX 4*, *TBS1*, *Vg*, *fox E4* or *fox A2,* and other potential candidates, which have previously been reported in vertebrates and were assumed to be present in mollusks. Some of these genes were validated by qRT-PCR and can be considered highly relevant to sex differentiation or determination. Among these candidate genes, *dmrt 1* is documented as a well-known sex determination and differentiation gene in vertebrates [16, 17]. Naimi et al. found that a *dmrt 1* gene named *Cg-DMl* was expressed in female and male oyster gonads, but that its expression in the testis was significantly higher than in the ovaries during late gametogenesis [18]. Therefore, the study speculated that *Cg-DMl* played a role in sex determination and differentiation, which is consistent with our results and with the results of Yang et al. [6]. Testis-specific expression of *Ha-DMRT 1* and ovary-specific expression of *Ha-VTG1* have also been validated through semiquantitative RT-PCR as important genes in the gonad development of *Haliotis asinine* [19].

Eighteen male-specific genes from the turquoise module, 12 genes highly expressed in males from the green module, nine female-specific genes from the coral1 module, and five genes highly expressed in females from the black module were selected in our study. These genes were all previously reported to be related to sex or gonad development. The *sox* genes are SRY (sex-determining region on Y chromosome) boxes, and they encode a group of proteins with a conserved HMG (high mobility group) domain which can bind and bend DNA to open the testis determination pathway directly and close the ovary pathway indirectly in mammalian species [20]. Some members of the *forkhead box* family are transcription factors essential for the early regulation of ovarian development, and Liu et al. discovered that Cf*-foxl 2* is mainly expressed in the ovaries in a sexually dimorphic pattern at the RNA level using semiquantitative RT-PCR [21]. *Pyfoxl 2* has also been found to be ovary-biased, and *Pydmrt* and *Pysox H* have been classified as testis-biased [9]. This is consistent with our results that *fox E4* and *fox A2* were ovary-biased, and that *dmrt 1* and *Sox 30* were testis-biased. However (Table 1), *sox 9* and *foxl 2* were not found to be sex biased in our RNA-Seq data, and this is worthy of further research. Furthermore, Ghiselli et al. determined that *Sox 30* was a good candidate gene for the differentiation of developing male germ cells in the manila clam (*Ruditapes philippinarum*), and that its function was differentiation of developing male cells [22]. *Cg-sox E* mRNA expression is not restricted to one sex, one type of germ cell and two early stages, Santerre et al. concluded that *Cg-sox E* was a new potential gene in the sex-determining pathway of the Pacific oyster (*Crassostrea gigas*), a hermaphroditic Lophotrochozoan [23].

**Table 1:** Primers for quantitative real-time PCR and gene annotations

Gene ID	Annotation	Sequence 5′ → 3′
ncbi_110441976	Transcription factor SOX-30	F GAAACTTTGATGACGAGGCG R GCTGACATTGAAGAATCATCGGT
ncbi_110441989	cAMP and cAMP-inhibited cGMP 3′,5′-cyclic phosphodiesterase 10A isoform X2	F GACTCCCAGCGGCATCTAA R CAAGGAACAGCGGTCAGCAT
ncbi_110444113	Leucine-rich repeat-containing protein 74B-like isoform X2	F GGTGTGTAAGAAGTTGGGTGTT R CGAACTTCCCAGACCGTG
ncbi_110450487	Doublesex- and mab-3-related transcription factor 1 isoform 1	F CCCCTAAATGTTGTCGCTGTC R TGAGGATAACGGTGTATGAGCA
ncbi_110463608	Transcription factor RFX4 isoform X3	F GTGATGACGGACCCTTGG R GGTTTACAATAGGGGCAGGC
ncbi_110445662	Protein B4	F GAGTCGCATCGTCTGTAAGGA R GCCGCTTTTCCTACCCTGA
ncbi_110450517	Cytochrome P450 1A4-like	F GCTTTACTTCCTCGCCCAAT R AACAGTAGGTCTCCGATGATGG
ncbi_110448784	Vitellogenin	F GCCCTTCTATGCGACTCAAT R TATCCATTCCTCTCAGGTAGTAG
XLOC_015112	Uncharacterized transcriptional regulatory protein TBS1-like	F TCACAGGGCAATGACGCAC R GCCCATCCTACTGACCCTG
β-actin		F CCAAAGCCAACAGGGAAAAG R TAGATGGGGACGGTGTGAGTG

Shi et al. (2018) put forward a sex reversal/differentiation pathway for *Chlamys nobilis*. They deduced that the significantly high expressions of *foxl 2*,*β-catenin*, and *sry* in intersex individuals provided preliminary evidence for possible interactions among these male- and female-promoting genes [24]. As upstream genes in sex determination/differentiation, *Wnt 6-like* and *Wnt 11B* were highly expressed in the female immature stage gonads, and*β-catenin* was highly expressed in the male immature stage gonads. These genes may have threshold values in the early gonad development stage which serve as a valve to control downstream gene expression.

In terms of male-related genes, leucine-rich repeat (LRR) proteins often mediate protein–protein interactions, and act as a scaffold to link signaling molecules, including PP1, to the manchette near potential substrate proteins important for spermatogenesis [25]. Recent studies have shown that the ovaries constantly repress male-specific genes from embryonic stages of development to adulthood [26]. The degree of polyglutamylation or hyperglutamylation serves as stabilizer and must be tightly controlled for the doublet stability of *MTs* [27]. WD repeat-containing proteins on the Y chromosome were first reported in the white campion (*Silene latifolia*), which is one of the few plants with separate sexes and with X and Y sex chromosomes, as for human XY-linked genes, the sex-linked plant loci encoding WD-repeat proteins are likely to be involved in cell proliferation, they cease recombining at different times and reveal distinct events in the evolutionary history of the sex chromosomes [28]. Expression of *tssk-3* is induced at puberty, persists during adulthood, and is restricted to the interstitial Leydig cells of mature male mice [29]. Giorgia et al. reported that cyclic nucleotides were involved in germ cell development and function, and discovered the presence of a calcium-calmodulin dependent PDE with high affinity for both cAMP and cGMP in male mouse sperm cells in addition to a calcium-calmodulin-independent PDA with high affinity for cAMP which controlled the sperm capacitation and motility [30]. *TBX4* and other clubfoot susceptibility genes increase predisposition to clubfoot in human males; variation in penetrance can be caused by their modifier(s) with sex-biased expression(s), resulting in different expression patterns in males versus females [31]. Yuan et al. reported that *mCTL1* mRNA was expressed in several mouse tissues such as the muscles, brain, heart, and testis [32]. Numerous heat shock proteins (HSP) and heat shock factors (HSF) are involved in a wide variety of physiological regulation processes and signaling pathways, exhibit a cell-type-specific expression pattern during spermatogenesis, and play crucial roles in germ cell development. The altered expression of HSP/HSFs may be responsible for abnormal germ cell apoptosis and subsequent impaired spermatogenesis [33]. All the above mentioned genes have been identified as male-specific genes.

Some female-specific genes were identified in our research, and were also observed and verified in other studies. CYP2AU1 signals were observed in follicular cells from female gonads of all different gonadic stages, while in males only the spermatic follicle cells of the wall in the pre-spawning stage showed this signal. This result indicates that the cytochrome P450 gene product was involved in reproduction [34]. Meanwhile, many female-related genes were also reported in a large number of studies. The polypeptide N-acetylgalactosaminyltransferase-like protein 5 (GALNTL5) is involved in male fertility, it might play an important role in a range of reproductive processes as well as in Hu sheep sperm motility and capacitation [35]. However, the peptide derived from mouse testis associated spermadhesin protein (AWN) was not glycosylated by either isoform of ppGaNTase, these differences suggest that multiple forms of ppGaNTase are required to optimally O-glycosylate multi-site substrates [36]. We observed that *GALNT4* was a female-specific gene in the coral1 module in our investigation. Transthyretin (TTR) is a major thyroid hormone-binding protein in amphibian tadpoles whose plasma mRNA and protein levels are altered during metamorphosis. TTR transcripts are more abundant in males than females, and *TTR* is partially regulated by transcription factor FoxA2 in *Xenopus laevis* [37]*.* Interestingly, *fox A2* was a female-specific gene in *P. yessoensis* according to our observations. Female-predominant expression of the mouse gene *Cyp2b9* may be responsible for sexually dimorphic expression, and forkhead box A2 (FoxA2) (hepatic nuclear factor 3β) had a large contribution to the promoter activity of *Cyp2b9* gene expression in both sexes, the differences in the expression of CYP2B9 mRNA between males and females might be regulated by FoxA2 protein, then sexually dimorphic secretion of growth hormone is involved in female predominant expression of these genes [38]. The Drosophila ovo gene encodes a putative transcription factor (Ovo) with TFIIIA-like zinc fingers. It is required for *Drosophila* female germ line maintenance and gametogenesis, and does not have a function in males or in somatic tissues [39]. Both in mice and in humans, the gonadotropin hormones play fundamental roles in the male reproductive system. The sole targets for follicle-stimulating hormone (FSH) in males are the Sertoli cells, while *E2F* and *GATA-1* are required for the Sertoli cell-specific promoter activity of the follicle-stimulating hormone receptor gene [40]. According to our RNA-Seq data, *GATA-1* (*GATA-type zinc finger protein 1*), *Vg* (*Vitellogenin*), *Fuc-T* (*N-acetylglucosaminide 3-α-L-fucosyltransferase*), and *TBS1-like* were female-specific genes. As a nonpolar molecular carrier and a storage protein, Vg can combine and transfer lipids, proteins, vitamin, and carotenoids to oocytes during oogenesis, it also participates in the host immune defense in the noble scallop *Chlamys nobilis* [41]. GDP-fucose: N-acetylglucosaminide 3-α-L-fucosyltransferase (Fuc-T) activities were found to be expressed in the Chinese hamster ovary cells; it is involved in the biosynthetic pathway for the fucosylation of polylactosamine sequences in glycolipids and glycol-proteins [42]. However, the function of *TBS1* has not been discovered in any organism.

Despite the identification of some candidate genes, sex determination pathways in mollusks remain elusive. In our study, we proposed that *dmrt 1* may have a leading function in the sex differentiation pathway of *P. yessoensis*, because *dmrt 1* is an upstream regulator in vertebrates and was highly expressed in immature male scallop gonads. Its expression was different from genes in turquoise, green, coral1, and black modules. The results of a chromatin immunoprecipitation (ChIP) analysis and an electrophoretic mobility shift assay (EMSA) have demonstrated that the male sex-differentiation factor *dmrt 1* positively regulates the transcription of the Nile tilapia sox 9b gene [43], this is a good evidence for the validity of our results. *Dmrt 1* may directly or indirectly activate male- and female-related genes to determine the direction of sex differentiation.

## Conclusion

WCGNA can facilitate identification of key genes for sex differentiation and determination. Using this method, 49 sex-related genes belonging to five modules were identified, and a hypothesized *P. yessoensis* sex determination and differentiation pathway was constructed. In this pathway, *Pydmrt1* may have a leading function.

## Methods

### Animal and tissue sampling

**Fig. 9:**
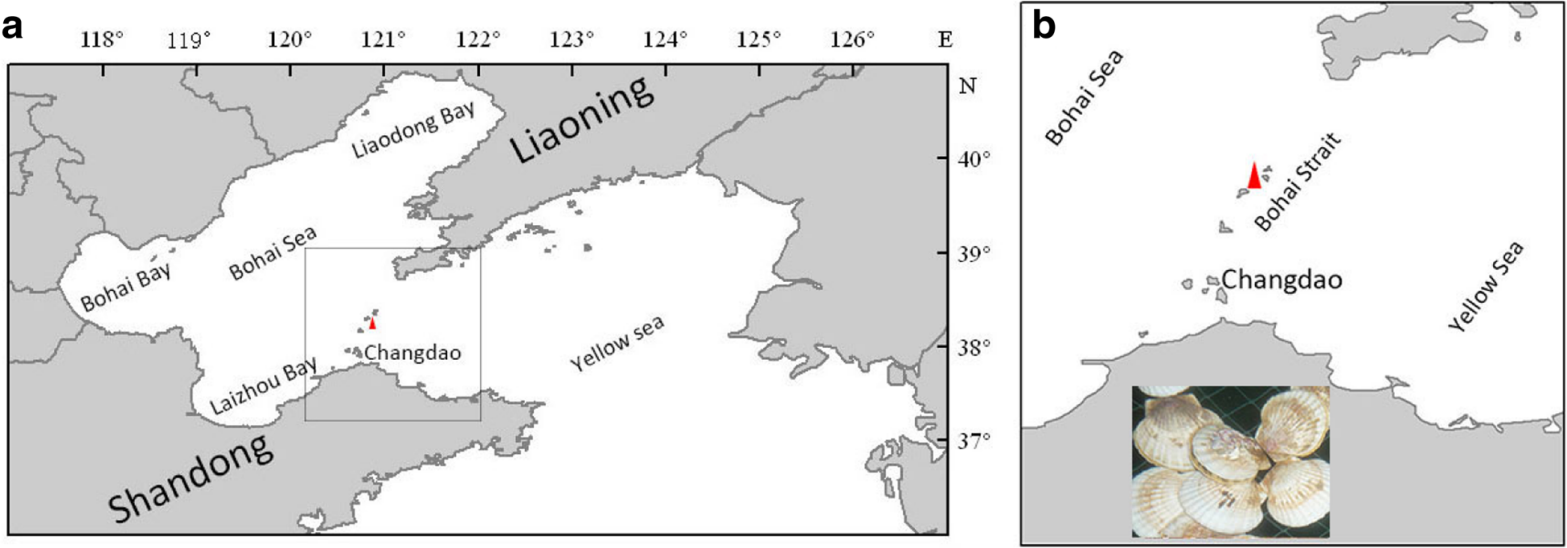
Sampling sites. The sampling map was created using ArcGIS V10.1(ESRI, CA, USA) and processed using Adobe Illustrator CS6(Adobe System Inc., San Francisco, CA, USA). Red triangle showed the sampling sites. Scallop photo in (b) belongs to Engineer Tao Yu

One- to 2-year-old Yesso scallops were collected monthly between January 2017 and July 2017 from Changdao, Shandong, China (Fig. 9). Gonad tissues were sampled from each scallop and were placed into liquid nitrogen for RNA extraction; a portion of each gonad was fixed for histological analysis. Other tissues were frozen and preserved separately in a − 80 °C freezer.

Biological traits such as shell length, shell height, shell width, shell color, body weight, shell weight, adductor muscle weight, and sex of each individual were recorded. Gonad development stage of the samples was identified and hermaphrodites were excluded using histological methods. In the immature stage (named PyUm), numerous oogonia or spermatogonia in the gonad follicles can be distinguished under a microscope through cellular morphology and size. In the mature stage (named PyM), gonads are completely filled with mature eggs or sperm cells. In the spawning stage (named PyO for females and PyS for males), numerous primary and mature gametes remain in the gonad follicles which become empty for spawning. Fifty-three animals were selected, and every two (or three or four) tissues from males or females were mixed into one sample. For convenience, every stage samples were assigned with the suffix “f” or “m” to distinguish samples from each sex. Thus, 18 samples were created and labelled as follows: PyUmf-1 to 3, PyUmm-1 to 3, PyMf-1 to 3, PyMm-1 to 3, PyOf-1 to 3, and PySm-1 to 3. Three biological repeats of male and female gonad tissues were used for RNA sequencing.

### RNA-Seq sequencing, gene expression analysis, novel gene identification

Total RNA was extracted from 18 samples using Trizol reagent (Invitrogen, USA), and RNA integrity was assessed using the Agilent 2100 Bioanalyzer (Agilent Technologies, Palo Alto, CA, USA). Eighteen cDNA libraries were constructed using 3 μg RNA (RIN>7) from each sample, following the conventional protocol. The prepared cDNA libraries were sequenced on Illumina HiSeqTM 4000 by Gene Denovo Biotechnology Co., Ltd. (Guangzhou, China).

Clean data were obtained after the adapters were removed from raw reads, and after reads with more than 10% unknown nucleotides and more than 50% low quality bases (Q value<20) were removed. The clean reads of each sample were then mapped to reference genome using TopHat2 (version 2.0.3.12) [44]. The read numbers mapped to each gene were counted via RMSE software [45]. The gene expression level was normalized using the FPKM method, and the expected number of fragments per kilobase of transcript sequence per million base pairs sequenced was calculated based on the length of the gene and the read count mapped to this gene. Then, reconstruction of transcript assemblies was conducted using the reference annotation-based transcripts (RABT) assembly program within the Cufflinks software package to obtain a comprehensive set of transcripts for further downstream differential expression analysis.

To identify sex-related novel transcripts, the reconstruction transcripts were aligned to the *P. yessoensis* genome and were divided into 12 categories using Cuffcompare. Genes with classcode “u,” lengths≥200 bp, and exon numbers≥2 were defined as novel genes, and were further annotated through alignment with the Nr and KEGG databases.

### Identification of differentially expressed genes (DEGs)

Differential expression analysis of groups among the six different gonadic categories was performed using the ‘edgeR’ package (http://www.r-project.org/). The resulting *P* values were adjusted to less than 0.05 to control the false discovery rate. Genes with ∣log2(FoldChange)∣>1 and a false discovery rate-adjusted *P* <0.05 were classified as differentially expressed genes. Differentially expressed gene (DEG) union was performed to assess the transcriptional pattern variations among the different gonadic categories, and a heat map of these DGEs was created using R scripts.

### Relationship analysis of samples

The correlation coefficient between two replicas was calculated to evaluate repeatability between samples. Principal component analysis (PCA) was performed using the ‘gmodels’ package in R (http://www.r-project.org/), and sample relationship analysis was presented via a heatmap.

### Weighted gene correlation network analysis (WGCNA)

To identify candidate genes and networks from sex differentiation DEGs, weighted gene correlation network analysis (WGCNA) was conducted to identify specific modules of co-expressed genes associated with each gonad development stage. We investigated the specific gene modules associated with the female and male gonad development stage separately. Prior to WGCNA, low-quality genes or samples (more than 50% were not expressed) were filtered out to improve the accuracy of the resulting network. To discover biologically or clinically significant modules, module eigengenes were used to calculate correlation coefficients with samples or sample traits. Intramodular connectivity of each gene was calculated and genes with high connectivity tended to be hub genes which might have important functions. The networks were visualized using Cytoscape_3.3.0. For genes in each module, gene ontology (GO) and KEGG pathway enrichment analyses were conducted to analyze the biological functions of modules. Significantly enriched GO terms and pathways in genes in a module compared to background genes and pathways were defined by a hypergeometric test and a threshold of false discovery rate (FDR) of less than 0.05.

Forty-nine hub genes related to gonad development were selected to serve as key regulators connected to a large number of nodes to construct co-expression networks based on modules or sex group data. Based on sex group samples, the following selection conditions were set: cor > 0.95, cor < − 0.6, and *p* < 0.05. Potential hub genes with sex differences in the transcript abundance were selected for real-time PCR.

### Validation of RNA-Seq data through quantitative real-time PCR assays

To further validate the confidence of the high-throughput transcriptome sequencing and the genes that play important roles in sex differentiation or determination, 15 differentially expressed genes were selected and analyzed via qPCR. The qRT-PCR analysis was performed with three biological and three technical replicates. The RNA samples conformed to the required purity criteria (A260/A230>2.0, and A260/A280 of 1.8–2.0), and the integrity of the RNA samples was assessed by agarose gel electrophoresis. Then, 1 μg total RNA samples were reverse transcribed into cDNA using the PrimeScript™ RT reagent Kit with gDNA Eraser (Takara, Japan). Quantitative real-time PCR was performed using SYBR® Premix Ex Taq (Takara) according to the manufacturer’s instructions on a Mx3000P (Agilent Stratagene, Agilent Technologies, Palo Alto, CA, USA) in 20-μl reactions. The PCR amplification procedure was carried out at 95 °C for 90 s, followed by 40 cycles at 95 °C for 5 s, 60 °C for 15 s, and 72 °C for 20 s; this was followed by disassociation curve analysis in an ABI 7500 fast real-time PCR system (Applied Biosystems, USA).

The *P. yessoensis* β-actin gene was used as an internal reference [6, 46]. The comparative Ct method (2^-△△Ct^ method) was used to calculate the relative gene expressions of the samples, which were normalized using the β-actin mRNA level. The expression data were subsequently subjected to independent t-tests in SPSS 18.0 to determine whether there were any significant differences at the *P* < 0.05 level. Fifteen sex-biased genes were randomly selected to design primers based on the NCBI database, nine gene primer pairs created using these genes are listed in Table 1. The fold changes of these genes in female versus male gonads were calculated via FPKM. The genes’ log2 fold change values of qRT-PCR and RNA-Seq were used for graphical presentation.

## Additional files

Additional file 1: **Figure S1**. Venn diagram of sex-biased genes. (PNG, 28 kb). (PNG 27 kb)

Additional file 2: **Table S1**. Forty-nine representative genes related to sex determination and differentiation in the transcriptome of *P. yessoensis*. (WORD, 36 kb) (DOCX 35 kb)

Additional file 3: **Figure S2**. Male-related gene co-expression networks for turquoise module. Red squares represent the hub genes ncbi_110441989 (*PDE*), ncbi_110442454 (*tssk-3*), ncbi_110466991 (*WD rcp*), and ncbi_110444113 (*LRR*) in the turquoise module. (PDF, 11 kb) (PDF 10 kb)

Additional file 4: **Figure S3**. Male-related gene co-expression networks for green module. Red squares represent the hub genes ncbi_110443603 (*CTL1*), ncbi_110465229 (*actin 5C*), and ncbi_110441893 (*bHLH*) in the green module. (PDF, 14 kb) (PDF 13 kb)

Additional file 5: **Figure S4**. Female-related gene co-expression networks for coral1 module. Red squares represent the hub genes ncbi_110465748 (*GALNT4*), ncbi_110454866 (protein ovo-like isoform X4), ncbi_110450517 (*CYP1A4-like*), ncbi_110450286 (*fox A2-like*), and XLOC_015112 (*TBS1-like*) in the coral1 module. (PDF, 10 kb) (PDF 9 kb)

Additional file 6: **Figure S5**. Female-related gene co-expression networks for black module. Red squares represent the hub genes ncbi_110456621 (*Fuc-T*), ncbi_110447244 (*collagen alpha-2(I) chain*), ncbi_110440232 (uncharacterized protein LOC105336037), and ncbi_110448784(*Vg*) in the black module. (PDF, 6 kb) (PDF 5 kb)

## Data Availability

The raw reads produced in this study were deposited in the NCBI SRA with the accession number SRP 199818 under Bioproject PRJNA544564.

## Abbreviations

*bHLH*: *Basic helix-loop-helix and HMG box domain- containing protein*
*Cg*: *Crassostrea gigas*
*CTL*: *Choline transporter-like protein*
DEG: Differentially expressed gene
*dmrt 1*: *Dsx and mab-3 related transcription factor 1*
*fox*: *Forkhead box*
FPKM: Fragments per kilobase of exon per million reads mapped
*Fuc-T*: *Glycoprotein 3-alpha-L- fucosyltransferase*
*GALNT*: *Polypeptide N-acetylgalactosaminyltransferase*
GO: Gene ontology
KEGG: Kyoto Encyclopedia of Genes and Genomes
*LRR*: *Leucine-rich repeat*
Mf: Mature females
Mm: Mature males
*MTs*: *Stabilizer of axonemal microtubules*
Of: Ovulation of females
PCA: Principal component analysis
*PDE*: *cAMP and cAMP-inhibited cGMP 3′,5′-cyclic phosphodiesterase*
*Py*: *Patinopecten yessoensis*
Sm: Sperm emission of males
*sox*: *Sry-type high mobility group box*
SRY: Sex-determining region on Y chromosome
*TBS 1*: *Transcriptional regulatory protein TBS1*
*TBX*: *T-box transcription factor*
TFs: Transcription factors
*tssk*: *Testis-specific serine/threonine-protein kinase*
Umf: Immature females
Umm: Immature males
*Vg*: *Vitellogenin*
*WD rcp*: *WD repeat-containing protein on Y chromosome-like*
WGCNA: Weighted gene co-expression network analysis
