# Supplementary figures and images for: Transcriptomics analysis revealing candidate genes and networks for sex differentiation of yesso scallop (Patinopecten yessoensis)
Source: BMC Genomics. 2019 Aug 23;20:671. doi: 10.1186/s12864-019-6021-6 (PMC6708199; doi:10.1186/s12864-019-6021-6)

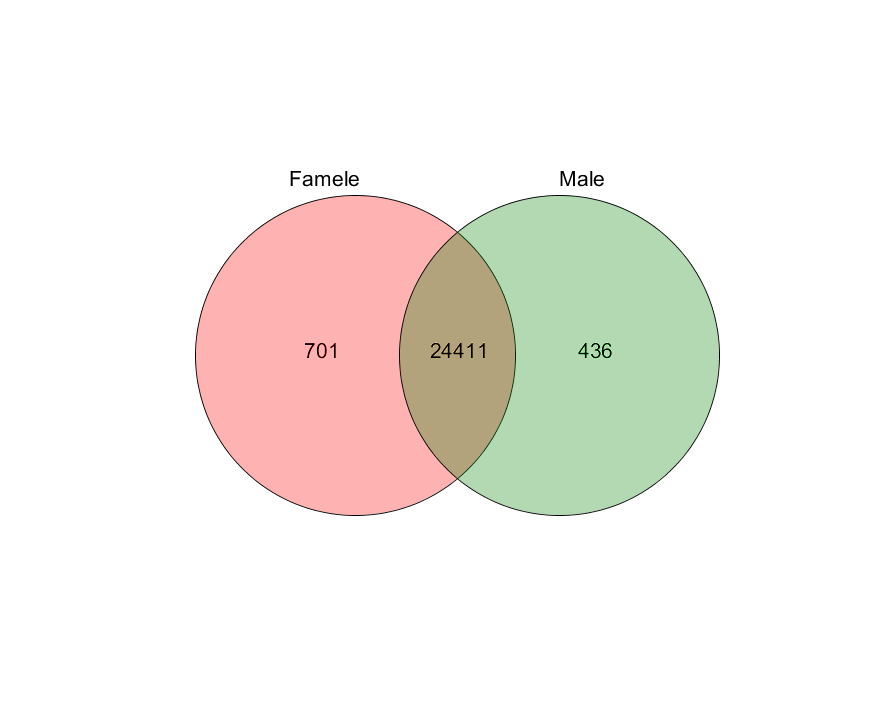

Supplement: Supplementary file 1 — Figure S1. Venn diagram of sex-biased genes. (PNG, 28 kb). (PNG 27 kb) [file 12864_2019_6021_MOESM1_ESM.png]

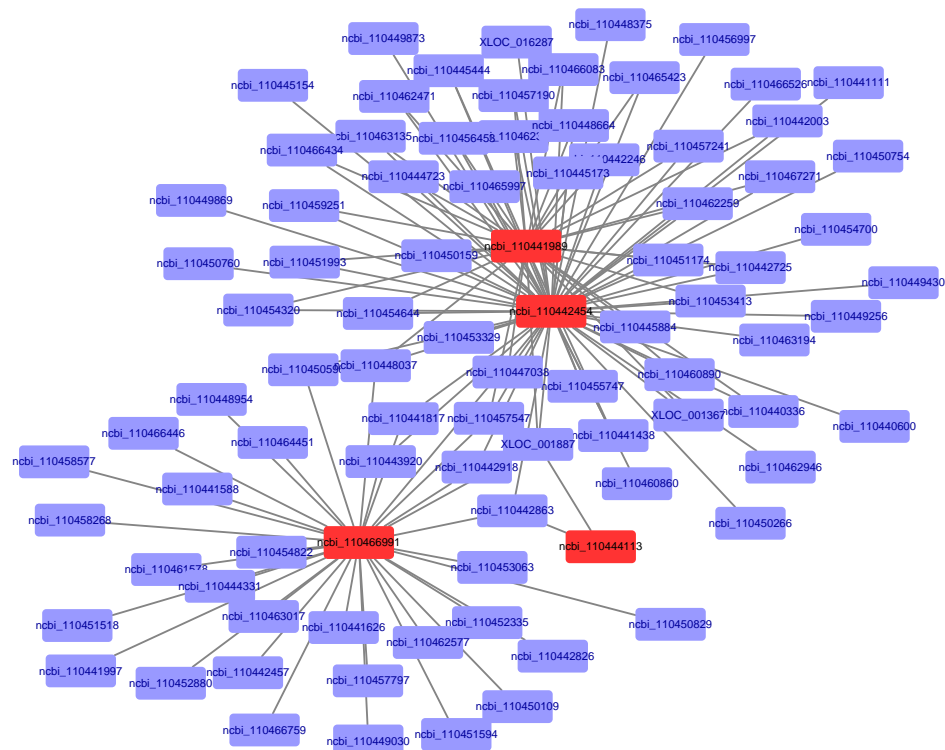

Supplement: Supplementary file 3 — Figure S2. Male-related gene co-expression networks for turquoise module. Red squares represent the hub genes ncbi_110441989 (PDE), ncbi_110442454 (tssk-3), ncbi_110466991 (WD rcp), and ncbi_110444113 (LRR) in the turquoise module. (PDF, 11 kb) (PDF 10 kb) [file 12864_2019_6021_MOESM3_ESM.pdf]

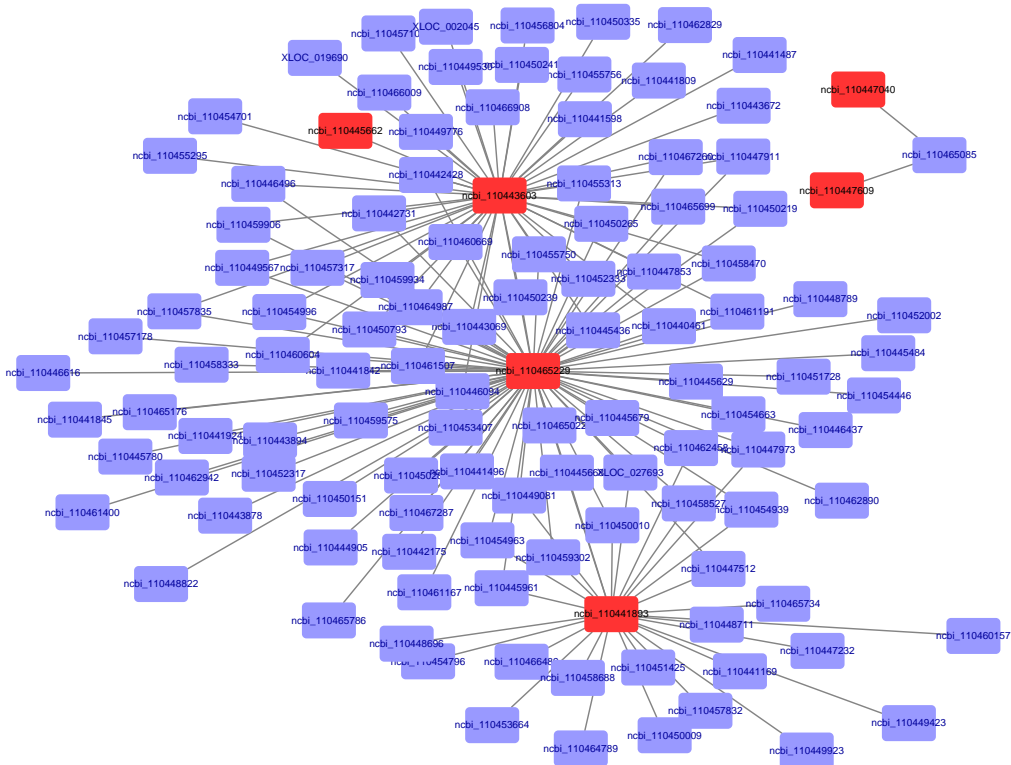

Supplement: Supplementary file 4 — Figure S3. Male-related gene co-expression networks for green module. Red squares represent the hub genes ncbi_110443603 (CTL1), ncbi_110465229 (actin 5C), and ncbi_110441893 (bHLH) in the green module. (PDF, 14 kb) (PDF 13 kb) [file 12864_2019_6021_MOESM4_ESM.pdf]

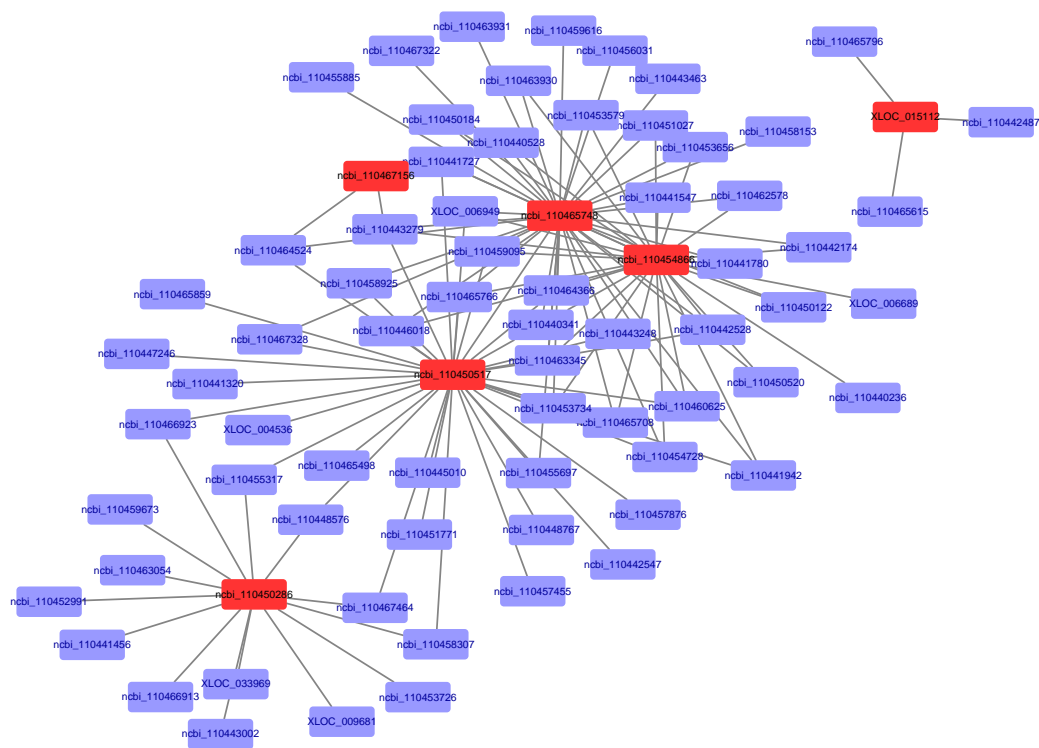

Supplement: Supplementary file 5 — Figure S4. Female-related gene co-expression networks for coral1 module. Red squares represent the hub genes ncbi_110465748 (GALNT4), ncbi_110454866 (protein ovo-like isoform X4), ncbi_110450517 (CYP1A4-like), ncbi_110450286 (fox A2-like), and XLOC_015112 (TBS1-like) in the coral1 module. (PDF, 10 kb) (PDF 9 kb) [file 12864_2019_6021_MOESM5_ESM.pdf]

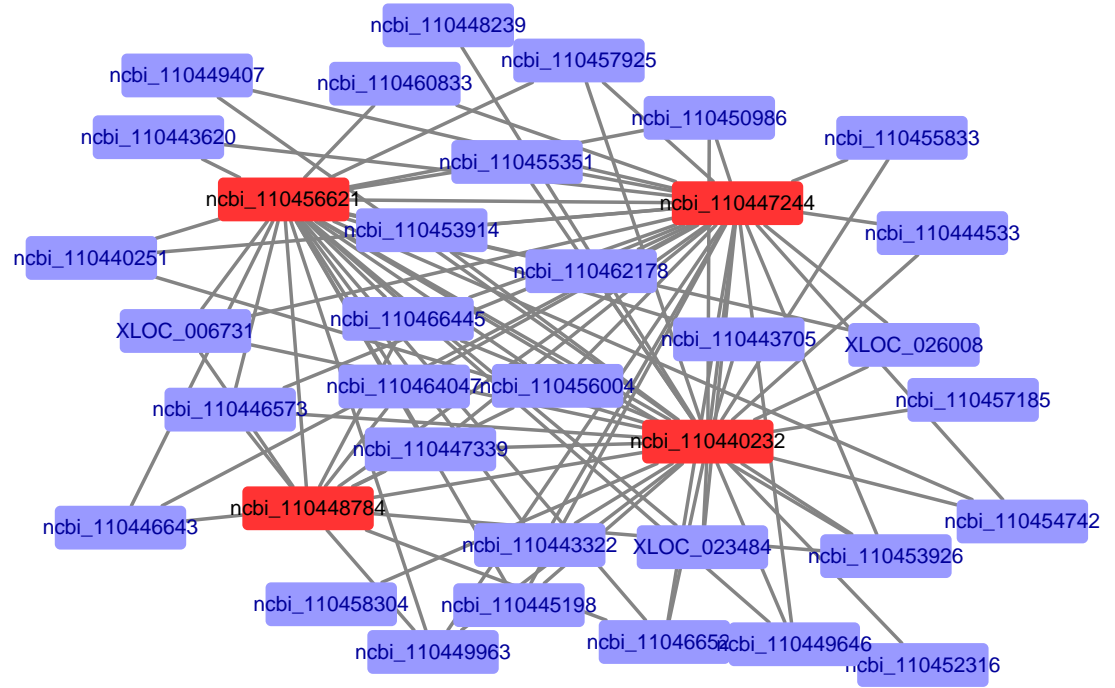

Supplement: Supplementary file 6 — Figure S5. Female-related gene co-expression networks for black module. Red squares represent the hub genes ncbi_110456621 (Fuc-T), ncbi_110447244 (collagen alpha-2(I) chain), ncbi_110440232 (uncharacterized protein LOC105336037), and ncbi_110448784(Vg) in the black module. (PDF, 6 kb) (PDF 5 kb) [file 12864_2019_6021_MOESM6_ESM.pdf]
