# Supplementary material for: Transcriptomics analysis revealing candidate genes and networks for sex differentiation of yesso scallop (Patinopecten yessoensis)
Source: BMC Genomics. 2019 Aug 23;20:671. doi: 10.1186/s12864-019-6021-6 (PMC6708199; doi:10.1186/s12864-019-6021-6)
Supplement: Supplementary file 2 — Table S1. Forty-nine representative genes related to sex determination and differentiation in the transcriptome of P. yessoensis. (WORD, 36 kb) (DOCX 35 kb) [file 12864_2019_6021_MOESM2_ESM.docx]

Table S1 Forty-nine representative genes related to sex determination and differentiation in the transcriptome of *P. yessoensis.*

| **Eighteen male- specific genes in turquoise module** | | | | | | | | | | | | | | | | | | |
| --- | --- | --- | --- | --- | --- | --- | --- | --- | --- | --- | --- | --- | --- | --- | --- | --- | --- | --- |
| Gene ID | PyUmf-1_fpkm | PyUmf-2_fpkm | PyUmf-3_fpkm | PyUmm-1_fpkm | PyUmm-2_fpkm | PyUmm-3_fpkm | PyMf-1_fpkm | PyMf-2_fpkm | PyMf-3_fpkm | PyOf-1_fpkm | PyOf-2_fpkm | PyOf-3_fpkm | PyMm-1_fpkm | PyMm-2_fpkm | PyMm-3_fpkm | PySm-2_fpkm | PySm-3_fpkm | Function |
| ncbi_110441976 | 0 | 0.84 | 0 | 16.75 | 25.52 | 44.03 | 0.06 | 0.14 | 0.03 | 0.04 | 0.05 | 0.05 | 16.2 | 72.12 | 32.8 | 63.21 | 43.2 | Transcription factor SOX-30 |
| ncbi_110444113 | 0.01 | 3.83 | 0.36 | 84.76 | 109.48 | 128.46 | 0 | 0 | 0 | 0 | 0.11 | 0 | 42.89 | 453.37 | 109.56 | 263.81 | 131.82 | leucine-rich repeat-containing protein 74B-like |
| ncbi_110442246 | 0.67 | 9.58 | 0.8 | 345.53 | 301.85 | 493.37 | 0.53 | 0.59 | 0.42 | 0.26 | 0.29 | 0.52 | 369.25 | 1722.53 | 379.7 | 1256.07 | 530.86 | stabilizer of axonemal microtubules 1 |
| ncbi_110456453 | 0.48 | 56.42 | 13.34 | 2022.34 | 2063.51 | 2788.22 | 1.58 | 1.56 | 0.71 | 0.66 | 1.04 | 1.13 | 2036.25 | 7867.89 | 2094.45 | 5458.83 | 2525.73 | arginine kinase-like |
| ncbi_110466991 | 0 | 1.75 | 0.02 | 30.44 | 36.67 | 53.26 | 0.01 | 0 | 0 | 0 | 0.01 | 0.01 | 39.81 | 190.95 | 60.15 | 137.56 | 67.08 | WD repeat-containing protein on Y chromosome-like |
| ncbi_110463608 | 0.54 | 1.62 | 0.26 | 8.36 | 10.62 | 14.8 | 0.55 | 0.24 | 0.36 | 1.98 | 0.72 | 1.05 | 23.85 | 49.57 | 19.87 | 33.16 | 21.49 | transcription factor RFX4 |

| ncbi_110448375 | 1.87 | 2.56 | 2.06 | 11 | 14.63 | 15.09 | 1.69 | 1.48 | 2.7 | 4.06 | 3.25 | 2.62 | 9.66 | 46.41 | 12.41 | 33.81 | 16.98 | transcription factor E2F8 |
| --- | --- | --- | --- | --- | --- | --- | --- | --- | --- | --- | --- | --- | --- | --- | --- | --- | --- | --- |
| ncbi_110442454 | 0.05 | 1.41 | 0 | 70.69 | 75.21 | 93.13 | 0.03 | 0 | 0 | 0 | 0.05 | 0 | 88.61 | 544.88 | 77.03 | 271.2 | 101.52 | testis-specific serine/threonine-protein kinase 3 -like isoform X2 |
| ncbi_110462577 | 0 | 0.23 | 0.13 | 13.73 | 7.34 | 9.91 | 0 | 0.07 | 0.06 | 0 | 0.06 | 0 | 5.66 | 56.1 | 6.82 | 37.01 | 4.76 | ATP-dependent Clp protease ATP-binding subunit clpX-like |
| ncbi_110467176 | 0.52 | 6.5 | 0.43 | 111.29 | 119.29 | 122.12 | 5.66 | 1.25 | 0.17 | 2.29 | 0.43 | 0.82 | 128.59 | 543.7 | 42.85 | 311.22 | 85.81 | Pyroglutamylated RFamide peptide receptor |
| ncbi_110445173 | 0.02 | 1.3 | 0.03 | 30.62 | 34.94 | 41.11 | 0 | 0.02 | 0 | 0.02 | 0.05 | 0 | 51.38 | 185.97 | 53.02 | 119.27 | 47.6 | biorientation of chromosomes in cell division protein 1 |
| ncbi_110458734 | 0.99 | 0.51 | 1.17 | 4.16 | 4.42 | 6.33 | 0.35 | 0.78 | 1.39 | 1.56 | 1.78 | 1.92 | 2.9 | 27.97 | 10.94 | 11.7 | 9.46 | Protein fem-1 like protein C |
| ncbi_110462517 | 21.96 | 20.14 | 20.14 | 61.01 | 76.23 | 63.12 | 10.35 | 9.04 | 8.98 | 24.14 | 18.82 | 13.34 | 82.46 | 215.87 | 123.36 | 85.33 | 60.82 | tumor protein p53-inducible nuclear protein 1 |
| ncbi_110441989 | 0.03 | 0.37 | 0.02 | 29.16 | 26.41 | 35.46 | 0.03 | 0.05 | 0.01 | 0.04 | 0.04 | 0.03 | 23.62 | 139.08 | 12.7 | 69.34 | 19.67 | cAMP and cAMP-inhibited cGMP 3',5'-cyclic phosphodiesterase 10A |
| ncbi_110465629 | 1.32 | 1.29 | 2.52 | 6.47 | 5.86 | 8.5 | 0.51 | 1.3 | 2.36 | 0.76 | 2.68 | 2.51 | 8.11 | 38.36 | 12.61 | 31.85 | 17.09 | forkhead box protein M1 |
| ncbi_110442133 | 0.47 | 21.81 | 0.63 | 429.31 | 411.5 | 705.94 | 0.45 | 0.13 | 0.12 | 0.08 | 0.46 | 0 | 536.81 | 2142.39 | 783.85 | 1485 | 972.63 | E3 ubiquitin-protein ligase MARCH3-like |
| ncbi_110463135 | 0 | 3.16 | 0 | 75.41 | 89.46 | 76.68 | 0 | 0 | 0 | 0.2 | 0.07 | 0.07 | 91.17 | 379.18 | 55.56 | 229.98 | 81.22 | translation initiation factor IF-2-like isoform X5 |
| ncbi_110445444 | 0.12 | 1.93 | 0.14 | 40.1 | 46.07 | 38.17 | 0.13 | 0.06 | 0 | 0 | 0.17 | 0.06 | 42.35 | 209.9 | 22.58 | 134.49 | 37.22 | Ras guanine nucleotide exchange factor Y |
| **Twelve high expression genes in male in green module** | | | | | | | | | | | | | | | | | | |
| Gene ID | PyUmf-1_fpkm | PyUmf-2_fpkm | PyUmf-3_fpkm | PyUmm-1_fpkm | PyUmm-2_fpkm | PyUmm-3_fpkm | PyMf-1_fpkm | PyMf-2_fpkm | PyMf-3_fpkm | PyOf-1_fpkm | PyOf-2_fpkm | PyOf-3_fpkm | PyMm-1_fpkm | PyMm-2_fpkm | PyMm-3_fpkm | PySm-2_fpkm | PySm-3_fpkm | Function |
| ncbi_110441893 | 1 | 2.02 | 1.72 | 13.04 | 14.68 | 17.98 | 0.22 | 0.21 | 0.63 | 0.45 | 1.15 | 0.52 | 10.66 | 18.14 | 17.79 | 13.8 | 14.45 | basic helix-loop-helix and HMG box domain- containing protein 1 |
| ncbi_110454119 | 0 | 0.32 | 0.18 | 1.79 | 1.78 | 1.93 | 0.06 | 0 | 0 | 0 | 0 | 0.06 | 0.94 | 1.92 | 1.89 | 1.54 | 2.77 | T-box transcription factor TBX4 |
| ncbi_110446297 | 0.71 | 12.67 | 10.15 | 43.27 | 36.56 | 65.73 | 2.07 | 1.5 | 1.07 | 0.82 | 1.09 | 1.21 | 12.41 | 24.47 | 21.72 | 35.78 | 55.2 | HMG box |

| ncbi_110461191 | 1.38 | 8.96 | 6.47 | 54.11 | 73.84 | 62.98 | 3.82 | 13.04 | 1.93 | 2.12 | 2.28 | 2.28 | 35.04 | 99.01 | 58.22 | 53.67 | 52.69 | testis-expressed sequence 12 protein |
| --- | --- | --- | --- | --- | --- | --- | --- | --- | --- | --- | --- | --- | --- | --- | --- | --- | --- | --- |
| ncbi_110446094 | 3.67 | 2.58 | 2.13 | 14.72 | 18 | 21.76 | 2 | 2.44 | 2.53 | 8.32 | 2.91 | 2.27 | 13.16 | 25.53 | 14.61 | 22.65 | 14.65 | probable ATP-dependent DNA helicase HFM1 |
| ncbi_110447040 | 3.82 | 10.11 | 5.94 | 25.86 | 25.2 | 38.11 | 2.45 | 2.12 | 13.41 | 2.06 | 6.07 | 5.61 | 11.89 | 26.45 | 38.8 | 29.48 | 37.34 | WD repeat and HMG-box DNA-binding protein 1 |
| ncbi_110442731 | 1.26 | 0.91 | 2.92 | 7.08 | 8.05 | 9.39 | 0.4 | 0.51 | 1.27 | 0.93 | 2.48 | 2.28 | 2.96 | 14.99 | 9.17 | 11.79 | 7.65 | WD repeat-containing protein 5-like |
| ncbi_110465229 | 5.41 | 265.96 | 191.03 | 1376.95 | 1678.51 | 1821.33 | 9.33 | 9.55 | 10.11 | 4.6 | 17.81 | 11.79 | 489.16 | 2109.95 | 1297.69 | 1692.07 | 1420.12 | actin 5C, isoform B |
| ncbi_110445662 | 0 | 0.96 | 0.87 | 8.62 | 13.05 | 11.68 | 0.46 | 0.08 | 0.14 | 0.19 | 0.07 | 0.4 | 10.14 | 15.65 | 2.7 | 4.97 | 11.62 | protein B4 |
| ncbi_110461167 | 16.91 | 17.04 | 20.43 | 57.34 | 73.55 | 85.98 | 6.48 | 11.12 | 19.14 | 9.97 | 19.96 | 17.57 | 44.68 | 117.72 | 59.27 | 94.82 | 60.8 | DNA topoisomerase 2-beta |
| ncbi_110443603 | 3.91 | 13.25 | 7.07 | 61.5 | 71.94 | 95.75 | 3.19 | 2.82 | 5.22 | 1.91 | 1.54 | 1.94 | 48.92 | 124.79 | 54.01 | 90 | 74.96 | choline transporter-like protein 1 |
| ncbi_110447609 | 0.24 | 9.39 | 7.26 | 34.7 | 27.77 | 57.31 | 0.41 | 0.95 | 0.16 | 0.15 | 0.21 | 0.63 | 17.77 | 44.49 | 58.69 | 36.65 | 48.03 | Heat shock factor protein- like |

| **Five high expression genes in immature male in darkgreen module** | | | | | | | | | | | | | | | | | | |
| --- | --- | --- | --- | --- | --- | --- | --- | --- | --- | --- | --- | --- | --- | --- | --- | --- | --- | --- |
| Gene ID | PyUmf-1_fpkm | PyUmf-2_fpkm | PyUmf-3_fpkm | PyUmm-1_fpkm | PyUmm-2_fpkm | PyUmm-3_fpkm | PyMf-1_fpkm | PyMf-2_fpkm | PyMf-3_fpkm | PyOf-1_fpkm | PyOf-2_fpkm | PyOf-3_fpkm | PyMm-1_fpkm | PyMm-2_fpkm | PyMm-3_fpkm | PySm-2_fpkm | PySm-3_fpkm | Function |
| ncbi_110450487 | 0.41 | 30.6 | 23.47 | 125.92 | 120.02 | 256.89 | 0.89 | 1.22 | 0.22 | 0.7 | 0.4 | 0.72 | 59.45 | 29.15 | 87 | 38.65 | 74.67 | doublesex- and mab-3-related transcription factor 1 |
| XLOC_012297 | 0.22 | 0.1 | 0.15 | 4.38 | 0.28 | 59.99 | 0.04 | 0.16 | 0.04 | 0.1 | 0.3 | 0.18 | 0.6 | 0.06 | 3.63 | 0.28 | 0.44 | GPX2 |
| ncbi_110452526 | 0.07 | 1.18 | 0.8 | 3.92 | 5.42 | 15.74 | 0.11 | 0.16 | 0.25 | 0 | 0.19 | 0.18 | 1.27 | 5.29 | 2.23 | 3.23 | 2.38 | FUT2 |
| ncbi_110466598 | 0.2 | 0.16 | 0.33 | 1.11 | 0.81 | 2.96 | 0.09 | 0.21 | 0 | 0.19 | 0.03 | 0.05 | 1.07 | 0.37 | 1.92 | 0.94 | 0.73 | Transposon Tf2-9 polyprotein |
| XLOC_029984 | 0 | 10.47 | 7.9 | 30.57 | 69.85 | 141.97 | 0 | 0.68 | 0 | 0 | 0 | 0 | 40.42 | 4.06 | 4.87 | 0.68 | 3.1 | hypothetical protein CGI_10014399 |
| **Nine female-specific genes in coral1 module** | | | | | | | | | | | | | | | | | | |

| Gene ID | PyUmf-1_fpkm | PyUmf-2_fpkm | PyUmf-3_fpkm | PyUmm-1_fpkm | PyUmm-2_fpkm | PyUmm-3_fpkm | PyMf-1_fpkm | PyMf-2_fpkm | PyMf-3_fpkm | PyOf-1_fpkm | PyOf-2_fpkm | PyOf-3_fpkm | PyMm-1_fpkm | PyMm-2_fpkm | PyMm-3_fpkm | PySm-2_fpkm | PySm-3_fpkm | Function |
| --- | --- | --- | --- | --- | --- | --- | --- | --- | --- | --- | --- | --- | --- | --- | --- | --- | --- | --- |
| ncbi_110450517 | 66.17 | 29.99 | 60.78 | 0.6 | 0.44 | 0.41 | 57.85 | 123.94 | 121.17 | 5.97 | 54.7 | 94.26 | 0.11 | 0.27 | 0.2 | 0.48 | 0.38 | Cytochrome P450 1A4-like |
| XLOC_015112 | 20.21 | 5.03 | 7.5 | 0.96 | 1.5 | 0.91 | 47.18 | 63 | 17.69 | 6.93 | 11.59 | 22.16 | 1.79 | 0.75 | 1.58 | 0.81 | 1.03 | uncharacterized transcriptional regulatory protein TBS1-like |
| ncbi_110463930 | 85.79 | 39.88 | 78.76 | 1.01 | 0.49 | 0.45 | 124.08 | 166.58 | 135.32 | 10.27 | 58.5 | 88.21 | 0.06 | 0.08 | 0.06 | 0.5 | 0.37 | polypeptide N-acetylgalactosaminyltransferase 1-like |
| ncbi_110465748 | 95.52 | 40.73 | 60.36 | 0.11 | 0.09 | 0.06 | 138.4 | 201.09 | 161.89 | 12.3 | 78.55 | 131.5 | 0.07 | 0.17 | 0.05 | 0.04 | 0.07 | polypeptide N-acetylgalactosaminyltransferase 4 |
| ncbi_110450286 | 14.89 | 1.81 | 4.83 | 0.02 | 0.06 | 0.02 | 15.77 | 21 | 41.9 | 6.59 | 20.07 | 23.84 | 0 | 0.08 | 0 | 0.02 | 0.02 | forkhead box protein A2-A-like |
| ncbi_110466595 | 19.15 | 9.78 | 15.08 | 0.54 | 0.26 | 0.26 | 65.3 | 50.03 | 31.41 | 5.03 | 14.24 | 25.58 | 0 | 0.14 | 0.71 | 0.09 | 0.09 | forkhead box protein E4-like |
| ncbi_110454866 | 32.66 | 13.47 | 21.5 | 1.1 | 0.75 | 0.52 | 42.99 | 81.71 | 59.72 | 13.89 | 37.38 | 40.92 | 0.25 | 0.31 | 0.42 | 0.83 | 0.32 | protein ovo-like |
| ncbi_110455885 | 5.76 | 2.7 | 4.59 | 0.7 | 0.62 | 0.46 | 10.17 | 9.65 | 9.33 | 0.95 | 3.91 | 6.3 | 0.18 | 0.48 | 0.62 | 0.42 | 0.3 | transcription factor AP-2-beta-like |
| ncbi_110467156 | 40.13 | 8.33 | 29.3 | 1.96 | 3.65 | 1.57 | 41.66 | 39.63 | 40.42 | 6.09 | 24.73 | 49.23 | 2.39 | 0.97 | 1.96 | 0.95 | 1.47 | GATA-type zinc finger protein 1 |

| **Five high expression genes in female in black module** | | | | | | | | | | | | | | | | | | |
| --- | --- | --- | --- | --- | --- | --- | --- | --- | --- | --- | --- | --- | --- | --- | --- | --- | --- | --- |
| Gene ID | PyUmf-1_fpkm | PyUmf-2_fpkm | PyUmf-3_fpkm | PyUmm-1_fpkm | PyUmm-2_fpkm | PyUmm-3_fpkm | PyMf-1_fpkm | PyMf-2_fpkm | PyMf-3_fpkm | PyOf-1_fpkm | PyOf-2_fpkm | PyOf-3_fpkm | PyMm-1_fpkm | PyMm-2_fpkm | PyMm-3_fpkm | PySm-2_fpkm | PySm-3_fpkm | Function |
| ncbi_110448784 | 113.02 | 22.54 | 21.8 | 0.36 | 0.49 | 0.72 | 359.81 | 2563.9 | 5666.4 | 2.96 | 1734 | 1004 | 3.66 | 1.18 | 0.71 | 0.79 | 0.34 | Vitellogenin |
| ncbi_110456621 | 0.39 | 0.47 | 0.37 | 0.14 | 0.06 | 0.07 | 0.78 | 1.15 | 4.09 | 0.19 | 1.55 | 1.32 | 0.06 | 0.27 | 0.29 | 0.07 | 0.24 | glycoprotein 3-alpha-L- fucosyltransferase A |
| ncbi_110450905 | 1.29 | 0.13 | 0.32 | 0 | 0 | 0 | 1.21 | 0.82 | 1.54 | 0.25 | 0.53 | 0.95 | 0 | 0 | 0 | 0 | 0 | A disintegrin and metalloproteinase with thrombospondin motifs |
| ncbi_110440232 | 102.51 | 46.65 | 57.45 | 0.57 | 0.45 | 0.69 | 121.59 | 209.65 | 500.64 | 4 | 140.3 | 214.37 | 0.35 | 1.2 | 0.39 | 1.54 | 0.54 | uncharacterized protein LOC105336037 |
| ncbi_110447244 | 1.36 | 0.45 | 0.61 | 0.09 | 0.07 | 0.08 | 0.73 | 3.15 | 12.16 | 0.25 | 4.31 | 5.11 | 0.09 | 0.04 | 0.05 | 0.05 | 0.07 | collagen alpha-2(I) chain |
